# Recent Advances in Tactile Sensory Systems: Mechanisms, Fabrication, and Applications

**DOI:** 10.3390/nano14050465

**Published:** 2024-03-04

**Authors:** Jianguo Xi, Huaiwen Yang, Xinyu Li, Ruilai Wei, Taiping Zhang, Lin Dong, Zhenjun Yang, Zuqing Yuan, Junlu Sun, Qilin Hua

**Affiliations:** 1 School of Integrated Circuits and Electronics, Beijing Institute of Technology, Beijing 100081, China; jzx0024@auburn.edu (J.X.); weiruilai@bit.edu.cn (R.W.); yuanzq@bit.edu.cn (Z.Y.); 2 School of Integrated Circuit Science and Engineering, Beihang University, Beijing 100191, China; yanghw@buaa.edu.cn (H.Y.); lixinyuy@buaa.edu.cn (X.L.); 3 Institute of Flexible Electronics, Beijing Institute of Technology, Beijing 102488, China; 4 Tianfu Xinglong Lake Laboratory, Chengdu 610299, China; zhangtaiping@xlll.cn; 5 Henan Key Laboratory of Diamond Optoelectronic Materials and Devices, Key Laboratory of Materials Physics, Ministry of Education, School of Physics and Microelectronics, Zhengzhou University, Zhengzhou 450001, China; ldong@zzu.edu.cn; 6 Hefei Hospital Affiliated to Anhui Medical University (The Second People’s Hospital of Hefei), Hefei 230011, China; yang.zhenjun.ahmu@foxmail.com; 7 Guangxi Key Laboratory of Brain-Inspired Computing and Intelligent Chips, Guangxi Normal University, Guilin 541004, China

**Keywords:** tactile sensors, mechanism, fabrication techniques, HMIs, robotics

## Abstract

Flexible electronics is a cutting-edge field that has paved the way for artificial tactile systems that mimic biological functions of sensing mechanical stimuli. These systems have an immense potential to enhance human–machine interactions (HMIs). However, tactile sensing still faces formidable challenges in delivering precise and nuanced feedback, such as achieving a high sensitivity to emulate human touch, coping with environmental variability, and devising algorithms that can effectively interpret tactile data for meaningful interactions in diverse contexts. In this review, we summarize the recent advances of tactile sensory systems, such as piezoresistive, capacitive, piezoelectric, and triboelectric tactile sensors. We also review the state-of-the-art fabrication techniques for artificial tactile sensors. Next, we focus on the potential applications of HMIs, such as intelligent robotics, wearable devices, prosthetics, and medical healthcare. Finally, we conclude with the challenges and future development trends of tactile sensors.

## 1. Introduction

Tactile perception is essential for tool use, communication, and interaction, and is a key means for humans to learn, remember, understand, and comprehend the world. Human tactile perception and recognition are mainly achieved by assessing the intensity, distribution, and pattern of stimulus events applied to the skin [1,2,3,4,5]. The specific process is as follows: after the mechanical receptors on the skin receive external stimuli, these are then encoded into nerve impulses. These pulses are processed by neurons and synapses using primary functions such as adaptation, filtering, amplification, and memory, and are then transmitted to the cerebral cortex, resulting in high-level functions such as classification, recognition, and perceptual learning. Such a complex tactile system enables humans to perceive tactile features and patterns through touch and interaction, and to complete complex interactive tasks, such as fine grasping, texture discrimination, and object recognition. Endowing artificial intelligence systems, such as robots, wearable devices, and prosthetic limbs, with tactile recognition functions can greatly improve both their ability to interact with unstructured environments, as well as their cognitive ability and intelligence level when manipulating unknown objects, and can help them complete more complex and fine tasks [6,7,8,9,10,11].

With the rapid development of functional materials, the research of tactile sensors has made great progress. Tactile sensors are not only used in the field of intelligent robots, but also can be used in clinical diagnosis, health monitoring, rehabilitation training, education, entertainment, and other fields [6,7,10,12]. The ideal artificial tactile device should have “skin-like” properties or even surpass the characteristics and functions of skin, such as being thin, flexible, stretchable, achieving morphological fusion (i.e., adapting the shape and structure at the interface of objects), high sensitivity, fast response ability, and so on [13,14]. The determination of these characteristics depends on reliable material synthesis, novel device structure design, and advanced fabrication techniques. Biologically, through perceiving neurons transmitting information received from mechanical receptors to the somatosensory cortex, the human brain processes these multidimensional signals through an energy-efficient and fault-tolerant computational process. Therefore, humans tend to remember frequent mechanical stimuli and establish sensory memory, short-term memory, and long-term memory, as well as human reflex responses to these stimuli. Neural plasticity, i.e., the evolutive ability of the brain throughout an individual’s lifetime, plays a key role in information processing [15,16,17]. Inspired by the neural system and its plasticity, researchers have developed neuromorphic electronic devices, including artificial synapses and artificial neurons [18,19,20,21,22], in which memristors can act as the building blocks of compute-in-memory for processing sensory information, to break through von Neumann architecture, and be given access to obtain a high operation speed and a low power consumption [6,23,24,25]. Artificial tactile sensors with memristor arrays can functionally mimic the neuromorphic intelligent interactions between human stimuli receptors and the central nervous system, such as threshold relaxation, non-adaptation, and sensitization, to sense and process touch information. In addition, the electrical connection between tactile sensors and neuromorphic devices can be used to mimic intelligent sensing and develop a tactile sensor that can communicate with the central nervous system, achieving a higher level of intelligent sensing.

However, there are still many application limitations of existing tactile sensors. Existing tactile sensors are far from reaching the resolution, sensitivity, and flexibility of human skin, which leads to information reception errors and the inability to accurately execute tasks when used for intelligent artificial systems [26,27,28,29]. Meanwhile, the rigidity of tactile sensors limits their applications on curved object surfaces, and then, an external power supply is required to drive the tactile sensors to work normally. This greatly increases both the energy consumption and the complexity of the system. Typically, the tactile sensors are spatially separated from the neuromorphic devices, limiting the development and application of highly integrated intelligent sensing networks [27,30,31,32,33]. Therefore, research into a biomimetic neuromorphic tactile sensing system coupled with intrinsic sensing mechanisms may be an effective solution.

**Figure 1:**
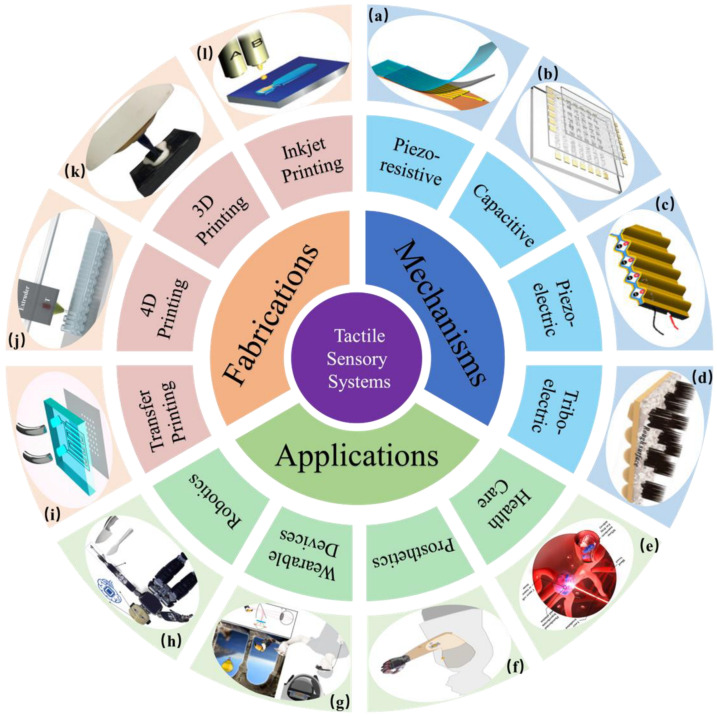
Schematic illustration of tactile sensors from mechanisms, fabrications, and applications. (**a**) Reproduced with permission from Ref. [34]. Copyright 2023, Springer Nature. (**b**) Reproduced with permission from Ref. [35]. Copyright 2018, Springer Nature. (**c**) Reproduced with permission from Ref. [36]. Copyright 2022, American Chemistry Society. (**d**) Reproduced with permission from Ref. [37]. Copyright 2021, Wiley. (**e**) Reproduced with permission from Ref. [38]. Copyright 2021, Science. (**f**) Reproduced with permission from Ref. [39]. Copyright 2023, Spinger Nature. (**g**) Reproduced with permission from Ref. [40]. Copyright 2022, Spinger Nature. (**h**) Reproduced with permission from Ref. [41]. Copyright 2020, Spinger Nature. (**i**) Reproduced with permission from Ref. [42]. Copyright 2023, Spinger Nature. (**j**) Reproduced with permission from Ref. [43]. Copyright 2021, Elsevier. (**k**) Reproduced with permission from Ref. [44]. Copyright 2023, Springer Nature. (**l**) Reproduced with permission from Ref. [45]. Copyright 2011, Springer Nature.

The architecture of this review paper is customized based on Figure 1 as follows: we summarize the advances of tactile sensors based on four types of mechanisms in the first part. Next, state-of-the-art fabrication techniques are discussed, including inkjet printing, 3D printing, 4D printing, and transfer printing. Part three focuses on the current and future application domains of intelligent robotics, wearable devices, and prosthetics. For the last part, we draw conclusions and present prospects with a wrap-up on the main theme presented in the review.

## 2. Mechanisms for Designing Tactile Sensors

**Figure 2:**
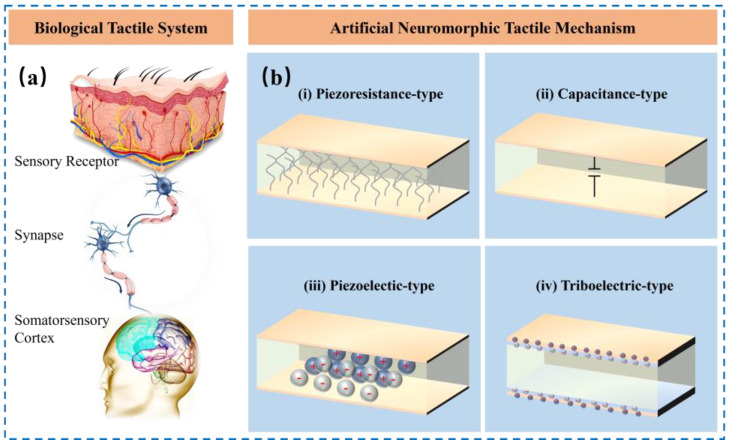
(**a**) The basic procedure of the synaptic current activated by external stimuli in the biological tactile system. (**b**) Schematic illustration of four typical working principles of flexible tactile sensor, (i) piezoresistive, (ii) capacitive, (iii) piezoelectric, and (iv) triboelectric.

Tactile sensation is one of the ubiquitous perception techniques in the human’s activity that collects information, such as stress, strain, shear stress, and vibration, of objects or other specific things via physical touch to help people adapt or respond to stimulus from the external environment. Flexible tactile sensors could be classified into four models based on different working principles, including piezoresistive, capacitive, piezoelectric, and triboelectric, which transform stimulus into electric signals, as shown in Figure 2.

### 2.1. Piezoresistive Tactile Sensors

**Figure 3:**
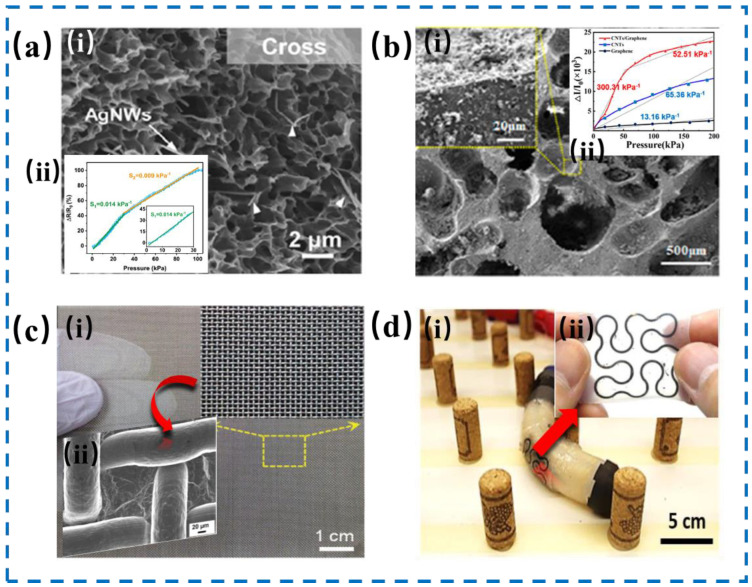
(**a**) Sectional view of porous AgNWs/PVDF composite (i) with the sensitivity under various pressure ranges (ii). Reproduced with permission from Ref. [46]. Copyright 2022, American Chemical Society. (**b**) SEM image of the porous PDMS with CNTs/graphene layer (i). (ii) Sensitivities of the flexible pressure sensor when coating by CNTs/graphene, CNTs, and graphene layers. Reproduced with permission from Ref. [47]. Copyright 2022, American Chemical Society. (**c**) SEM images of PDMS/CNT microstructures (ii) molded by the stainless-steel mesh (i), which was treated as a frame for PDMS/CNT microstructure. Reproduced with permission from Ref. [48]. Copyright 2020, Wiley. (**d**) Schematic illustration of the soft robotic earthworm, which was moving through a cylinder array (i). (ii) Photograph of serpentine piezoresistive strain sensors. Reproduced with permission from Ref. [49]. Copyright 2020, American Chemical Society.

Piezoresistive tactile sensors primarily rely on the piezoresistive effect of materials, that is, converting mechanical signals into electrical signals via changes in materials’ resistivity, contact resistivity, and quantum tunneling effects, and have gained widespread attention due to their simple structure, low energy consumption, and broad sensing range. So far, researchers have extensively studied high-performance piezoresistive tactile sensors on materials such as metal-based materials and carbon nanomaterials and structures like mesh and serpentine structures. As shown in Figure 3a, Chen et al. [46] developed a flexible piezoresistive sensor using a sensing layer composed of silver nanowires (AgNWs) and Polyvinylidene Fluoride (PVDF) composites, showcasing a unique pilotaxitic 3D porous architecture with evenly distributed AgNWs. This structure results from the synergistic effect of the N-Methyl pyrrolidone solvent and a water-based coagulation bath acting on the AgNWs/PVDF precursor. The sensor demonstrates exceptional sensitivity, measuring 0.014 kPa^−1^ in the 0 to 30 kPa pressure range and 0.009 kPa^−1^ for pressures from 30 to 100 kPa. In Figure 3b, Liu et al. [47] designed an ultra-sensitive flexible pressure sensor featuring carbon nanotubes (CNTs)/graphene on a porous Polydimethylsiloxane (PDMS) substrate. The substrate’s uniform microporous structure is created through microwave heating, offering a vast surface area for the application of conductive nanomaterials. This design results in remarkable pressure sensitivity, achieving approximately 300.31 kPa^−1^ in the 0 to 50 kPa range and 52.51 kPa^−1^ for pressures between 50 to 200 kPa. Apart from the material, the microstructure of the device significantly influences the performance of piezoresistive tactile sensors. As shown in Figure 3c, Arias et al. [48] produced a high-performance biomimetic skin pressure sensor using a mesh-molded periodic microstructure fabricated by peeling off from polystyrene (PS) template, in which a conductive layer of carbon nanotubes was uniformly spray-coated onto the inversely micro-structured PS template. And then, a layer of PDMS precursor was cast over it. This induced a low operating voltage of 1 V, high sensitivity of 20.9 kPa^−1^, and low detection limit of 7.4 Pa. In addition, Yeo et al. [49] introduced a flexible and stretchable wireless strain sensing system, involved in a highly adaptable strain sensor based on carbon nanomaterials and silicone elastomer, designed to control a robotic earthworm’s movements through a feedback loop in Figure 3d. This serpentine-shaped strain sensor shows stretchability of 100% and gauge factor of 38 and can help monitor normal movements of the robotic earthworm under the aid of nanomembrane interconnectors and wireless electronics. These specific materials and structures can pave an effective pathway for piezoresistive tactile sensors with high sensitivity, high accuracy, and low detection limits, making them very attractive for future advancement of human–machine interfaces.

**Figure 4:**
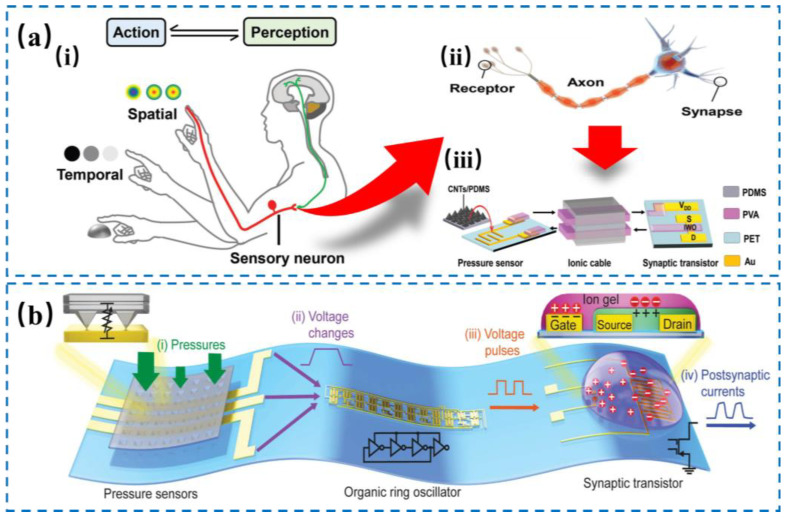
Schematic illustration of the fabrication of neuromorphic tactile systems. (**a**) The NeuTap concept design. (i) The fusion of spatial and temporal pattern recognition within the tactile perception-feedback loop. (ii) The structure of a sensory neuron’s function. (iii) The illustrative details for the intricate working process and components of the NeuTap system. Reproduced with permission from Ref. [55]. Copyright 2018, Wiley. (**b**) The structure of an artificial afferent nerve system involves pressure sensors, an organic ring oscillator, and a synaptic transistor for signal transduction, in which the single ring oscillator is connected to a synaptic transistor. The architecture allows for the integration of multiple ring oscillators, each linked to a cluster of pressure sensors, converging on a solitary synaptic transistor. Reproduced with permission from Ref. [56]. Copyright 2018, Science.

A typical neuromorphic tactile sensory system consists of a resistive pressure sensor and a neuromorphic device, corresponding to the mechanical receptor and the neuromorphic information processing in the biological system [50,51,52,53,54]. The most widely studied resistive pressure sensing unit is PDMS coated with a conductive layer. For example, Zhu and Chen et al. [55] created a low-error resistive neuromorphic tactile system (0.4%) that consisted of a carbon nanotube-based micro-structured resistive pressure sensor, an ionic wire, and an electrolyte-gated tungsten oxide transistor, as shown in Figure 4a. The pressure sensor’s resistance changes when external force is applied, which cause the voltage drop on the ionic wire. This affects the voltage pulse that goes to the neuromorphic transistor and leads to different channel modulation outcomes of the transistor. The ion migration produces a strong electric field that modulates the PSC by changing the carrier density within the channel. When the pressure is constant, more ions gather at the interface, increasing the PSC amplitude. When the pressure is removed, the voltage drop of the ionic wire drops quickly, the ions will slowly move back to their balance position, and the PSC will slowly go back to the initial level. The system can use the electrical response to external stimuli/release signals to recognize and extract the tactile features of the application.

Moreover, Bao and Lee et al. [56] integrated a carbon nanotube-based pressure sensor, a ring oscillator, and an organic electrochemical transistor (OECT) to construct an artificial afferent nerve, as shown in Figure 4b. In this system, the pressure signal could be converted into a voltage signal with frequency characteristics by the ring oscillator, which was used to simulate the mechanical receptor and the artificial nerve fiber. The electrical signal from the artificial nerve fiber is converted into PSC by the OECT neuromorphic transistor. By analyzing the frequency information of the electrical signals from multiple resistive mechanical receptors, the artificial afferent nerve could be used to achieve shape recognition, motion detection and braille recognition in simple scenarios. Moreover, the PDMS-based stretchable neuromorphic tactile system can exhibit all-round perception with only 50% loss compared with the initial value under tension.

### 2.2. Capacitance-Based Tactile Sensors

**Figure 5:**
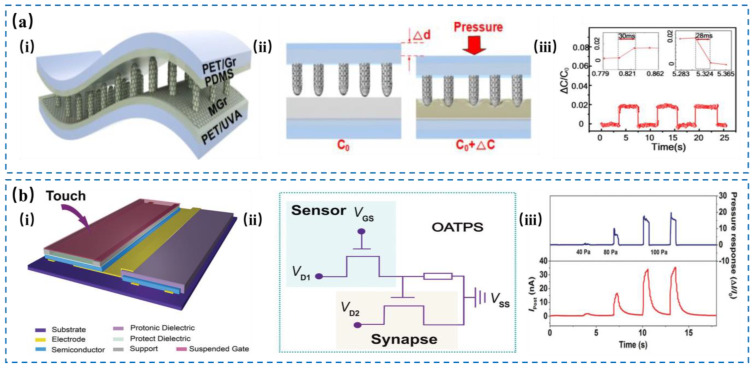
Structural illustration of capacitive tactile sensors. (**a**) A flexible pressure sensor based on μ-graphene electrodes. (i) The structure of MGr-based pressure sensor. (ii) Sensing mechanism of MGr-based pressure sensor. (iii) The response time of MGr-based pressure sensor. Reproduced with permission from Ref. [59]. Copyright 2019, American Chemical Society. (**b**) The structure design of the DOT-TPS (i). (ii) Equivalent circuit of the DOT-TPS. (iii) The relative changes of I_post_ and the response time for DOT-TPS when applying different pressures. Reproduced with permission from Ref. [63]. Copyright 2017, Wiley.

A capacitive tactile sensor is a mechanical receptor based on capacitance with pressure sensing function and memory with signal processing function, which can realize various functions of signal transduction and information processing [57,58,59,60,61,62]. The capacitance of the pressure sensor changes with the applied pressure and is converted into a pulse electrical signal, which is transmitted to the presynaptic end of the second neuromorphic transistor through the gate. When the presynaptic end receives the pulse signal, the migration of ions in the dielectric layer affects the accumulation state of charges in the organic semiconductor channel, thus modulating the postsynaptic current (PSC) flowing between source and drain. The characteristics of the PSC response depend on the amplitude, frequency, and duration of the applied pressure. Therefore, by detecting the changes of PSC in the system, dynamic pressure signals, time-varying signals and comprehensive tactile information can be collected and analyzed. A pioneering study integrated an organic field-effect transistor (OFET) that could sense transient pressure stimuli with another capacitive tactile OFET, and constructed a capacitive tactile device based on dual OFETs [59,63], as shown in Figure 5. Figure 5a describes one sensor with its unique layered design including a micro-structured graphene base, a PDMS middle layer, and a smooth graphene top layer, which could quickly react to pressure changes faster than human skin and traditional sensors [59]. Its quick response and recovery times were attributed to the design that efficiently alters capacitance with pressure changes, offering rapid and reliable sensory feedback, which made it more responsive than conventional tactile sensors limited by slower, viscoelastic responses. In Figure 5b, the dual-organic-transistor-based tactile perception element (DOT-TPE) system, designed to simulate human tactile perception, integrates synaptic OFETs with OFET-based pressure sensors [63]. This system translates tactile stimuli into electrical signals through pressure sensors, which are then processed by synaptic OFETs for cognitive tactile emulation. It features a suspended-gate OFET for pressure detection and a synaptic OFET for signal processing, effectively mimicking human touch sensitivity. Upon applying pressure, the device’s capacitance changes, leading to conductivity modulation and the generation of electrical spikes that are analyzed for dynamic tactile information. Testing with dynamic pressure cycles (40–100 Pa) showed that the device’s output current peaks with each stimulus, demonstrating its capability to replicate human tactile sensation.

### 2.3. Piezoelectric Tactile Sensors

**Figure 6:**
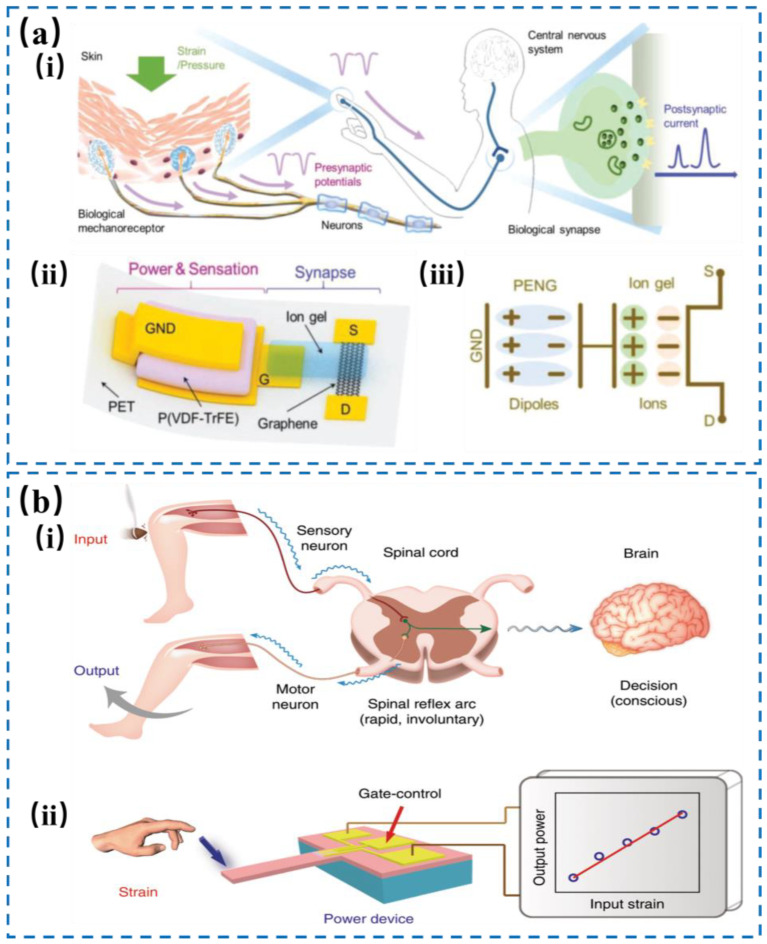
(**a**) The human sensory nervous system features mechanoreceptors in the skin that transform mechanical stimuli into presynaptic potential signals, which are then transmitted to the central nervous system via neurons and synapses (i). (ii) Schematic diagram of the graphene artificial sensory synapse based on piezotronics effect. (iii) An equivalent circuit diagram of the piezoelectric potential’s interaction through an ion gel. Reproduced with permission from Ref. [70]. Copyright 2019, Wiley. (**b**) In the human reflex arc, stimulation of a thigh muscle induces a response where the sensory neuron communicates an action potential to the spinal cord’s gray matter. Within the spinal cord, this neuron forms a direct synaptic link with a motor neuron. If the stimulus is sufficiently intense, it can initiate action potentials in the motor neuron, resulting in a knee jerk (i). (ii) The Strain-Powered Device (SPD) uses an external strain on a cantilever mechanism to mimic the logical processing of a human reflex, modulating output power based on mechanical input. Reproduced with permission from Ref. [72]. Copyright 2020, Springer Nature.

Specific materials with non-centrosymmetric structure (such as piezoelectric ceramics, Wood’s structure and piezoelectric polymers) will induce piezoelectric potential after applying pressure. The piezoelectric potential can be transmitted to the neuromorphic terminal, and a piezoelectric neuromorphic tactile system can be constructed to perceive spatiotemporal tactile information [36,64,65,66,67,68,69]. As shown in Figure 6a, a piezoelectric graphene artificial tactile system consisting of sensing, transmission and processing units was constructed by combining piezoelectric nanogenerators (PENG) and electrolyte-gated graphene transistors [70]. During mechanical deformation, the dipoles in the piezoelectric polymer were aligned in a uniform direction, inducing the generation of piezoelectric potential and promoting PENG to work as a self-powered mechanical receptor [71]. Under the influence of the piezoelectric potential, the ions in the electrolyte or dielectric would migrate and redistribute, to regulate the conductivity of the graphene channel and induce PSC response. The relationship between the piezoelectric polarization caused by mechanical stimulation and the transmission characteristics of the semiconductor endows PSC with the time and space information of external stimuli. The unique ion response behavior and strong ion/electron coupling phenomenon of the graphene/ion gel interface lay the foundation for biomimetic neural and biochemical sensing applications. Inspired by the biological reflex mechanism in Figure 6b, Wang et al. [72] proposed an artificial tactile system based on wood’s structure and GaN-based piezoelectric effect. The system used a cantilever structure to achieve pressure sensing, greatly simplifying the system complexity. When the cantilever beam was subjected to strain, the piezoelectric potential was produced, which would change the concentration of the 2D electron gas on the interface of AlGaN/AlN/GaN heterostructure. In this way, the piezoelectric potential can modulate the electron transmission in the neuromorphic transistor, thereby simulating the biological reflex process.

### 2.4. Triboelectric Tactile Sensors

**Figure 7:**
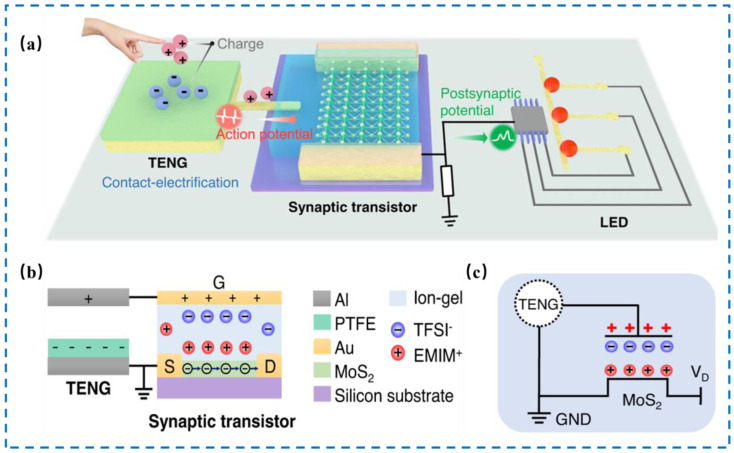
(**a**) Schematic illustration of triboelectric tactile systems activated by contact electrification, which includes a self-activation component, a synaptic transistor, and a functional circuit. (**b**) Schematic diagram of the CE-activated MoS_2_ synaptic transistor with annotations of each component. (**c**) Equivalent circuit of the CE-activated artificial afferents. Reproduced with permission from Ref. [84]. Copyright 2021, Springer Nature.

The mechanical displacement of triboelectric nanogenerators (TENG) can generate friction potential based on friction electrification and electrostatic induction. Combining friction potential with semiconductor devices, the carrier transportation of the semiconductor channel can be directly controlled by mechanical stimulus. The integration of TENG tactile sensors with Extended Gate Field Effect Transistor or EGFET [73,74], Ferroelectric Field Effect Transistor or FeFET [75,76,77], Floating-Gate Field Effect Transistor or FGFET [78,79,80], Semi-Floating-Gate Field Effect Transistor or SFGFET [81] and other neuromorphic transistors [82,83] has been used to develop self-powered tactile perception systems. The external force applied on the TENG is converted into voltage spikes, which are then captured by the neuromorphic transistor, producing PSC responses. In addition, in view of the self-powered nature of the TENG, the system greatly reduces energy consumption and demonstrates the spatiotemporal recognition ability of external stimuli. The artificial perception can be used to establish dynamic logic, as well as to identify the frequency/amplitude of external motions. As shown in Figure 7, Wang et al. [84] developed a neuromorphic tactile system consisting of TENG tactile sensors and MoS_2_-EGFET, in which the TENG mechanical receptor could be designed into different working modes, monitoring disparate tactile information, including mechanical displacement, lateral sliding motion, tactile signal, and pressure. The friction electric signal could activate the EGFET, which further enables the system to recognize spatiotemporal touch patterns by triggering the corresponding LED array to display the result. This can be used to realize visualization of virtual stimuli in the cerebral cortex.

So far, giant advances in neuromorphic tactile systems have been achieved. Here, Table 1 compares the specialties of neuromorphic tactile systems based on four types of mechanisms in these aspects, including sensor features, sensitivity, sensing range, and applications. By comparison, it was clear that each type offers distinct advantages and limitations based on their working principles and material properties. Resistive sensors are valued for their simplicity and robustness, while capacitive sensors excel in sensitivity and spatial resolution. Piezoelectric sensors stand out for their ability to generate voltage from mechanical stress, offering self-powering capabilities. Triboelectric sensors, on the other hand, leverage contact electrification and electrostatic induction, providing high sensitivity and the potential for energy harvesting. The choice among these sensors depends on the specific application requirements, including sensitivity, power consumption, flexibility, and environmental robustness.

## 3. Fabrication Techniques for Tactile Sensors

The fabrication techniques for neuromorphic tactile sensors determine their performance, functionality, and compatibility with different applications. So far, the mainstream fabrication techniques include inkjet printing, 3D printing, 4D printing, and transfer printing.

### 3.1. Inkjet Printing

**Figure 8:**
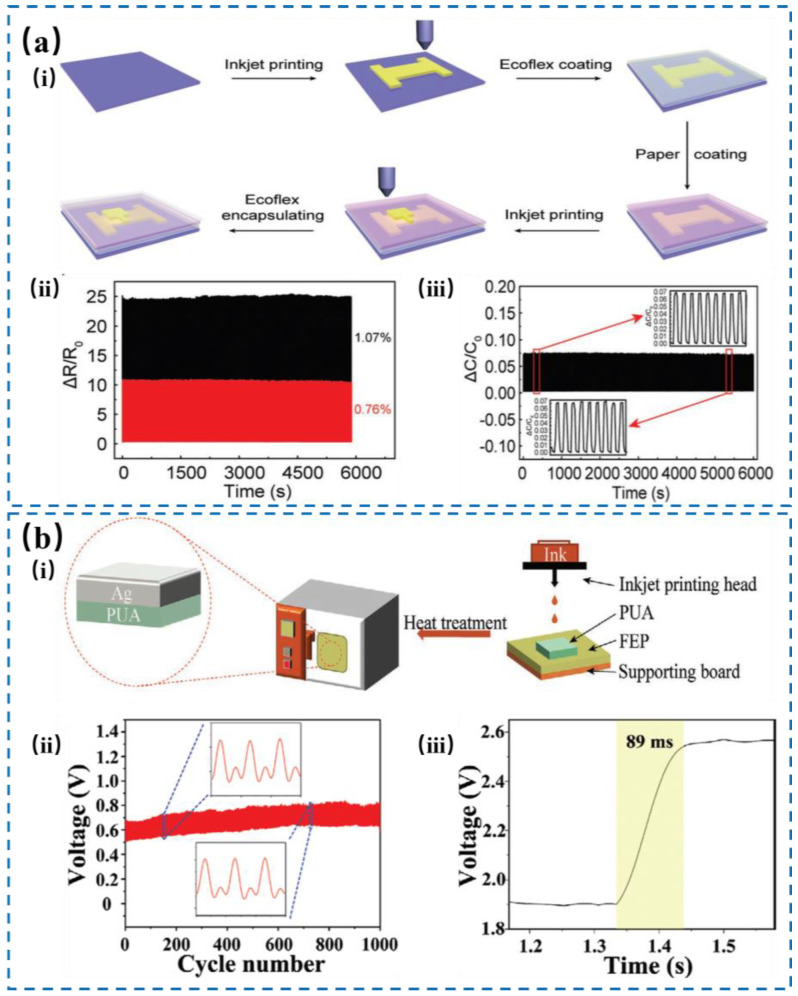
(**a**) Schematic illustration of bimodal sensor fabricated by inkjet printing (i). (ii) The durability of the strain sensor at a strain of 0.76% and 1.07%, respectively. (iii) The durability of the pressure sensor at a pressure of 202.8 kPa. Reproduced with permission from Ref. [96]. Copyright 2019, Wiley. (**b**) Schematic illustration of the fabrication process of PUA/Ag composite film as flexible electrode (i). (ii) Durability under 1% strain with a frequency of 0.1 Hz and 1000 stretching/releasing cycles. (iii) The response time of single-layer PUA/Ag-based strain sensor under 1% strain. Reproduced with permission from Ref. [97]. Copyright 2021, Wiley.

As an emerging technology, inkjet printing is receiving increasing attention for flexible electronics with superior digital capability, simple operating procedures, and high depositing resolution [93,94,95]. In addition, the merits of time efficiency and low cost help inkjet printing as a potential candidate for batch production of flexible electronics, which is beneficial for applications such as robotics, human motion monitoring, etc. Figure 8a shows a bimodal e-skin designed by Pan et al. [96] that can sense both strain and pressure using an inkjet-printing method, which used a microcracked metal film as a common electrode for both sensors without interfering with each other. The device had a high sensitivity of ~2 Pa for pressure sensor and stability with over 5700 cycles for pressure sensor and 4500 cycles for strain sensor and could detect the shape and pressure of various objects. In addition, no interfering signal between the pressure sensor and strain sensor was observed. To deal with the challenge of sensitivity and stability, Sun et al. [97] prepared a flexible sensor with a flat electrode and micro-structured electrode of metallic Ag nanoparticles on polyurethane acrylate (PUA) substrate via inkjet printing, as depicted in Figure 8b. The as-synthesized single-layer sensor showed an ultrahigh sensitivity with a gauge factor of >1100, durability of >1000 cycles under stretching and releasing, and response time of ~90 ms, while double-layer sensor with PUA/Ag composite films achieved a faster response capability for small pressure of 250 Pa.

### 3.2. Three-Dimensional (3D) Printing

Three-dimensional (3D) printing is a potential method to create one device rapidly through breaking up a 3D model into several slices in combination with computer design. The core point of 3D printing is materials, for which polymers, liquid metals, silver nanowires, carbon nanotubes, graphene, and other materials are the most commonly used in conductive flexible composite materials. Zeng et al. [98] developed a new type of piezoresistive sensor based on one ink composed of polydimethylsiloxane microspheres and carbon nanotubes via 3D printing to mimic the texture and sensitivity of human skin. As-prepared sensors were sensitive to applied shear stress with a sensitivity of ~2.08 kPa^−1^ at a pressure of 0.12 kPa and had a fast response time of 50 ms and high durability of over 8000 cycles. Utilizing the high mobility of graphene, Zeng et al. [99] designed one tactile sensor for microstructure perception using graphene-polydimethylsiloxane microspheres using 3D printing. Some properties of the flexible tactile sensor with complex functions were achieved, including a robust tensile capability with a stretch rate of 70% and a response time of 60 ms. Moreover, the sensitivity was up to 2.4 kPa^−1^, and the stability over 2000 cycles, which could be used to accurately measure pressure and distinguish the delicate morphology of surfaces. On one hand, the overall resistance of the conductive material is large due to the low CNT or graphene content added. On the other hand, the addition of dispersant will greatly affect the overall mechanical properties of the composite material. More importantly, the dispersants have certain toxicity, which is not friendly for the preparation of sensors. Therefore, most of the existing dermoid perception materials require complex molecular design or multi-step functionalization. The materials doped with conductive fillers often have a contradiction between printability and conductivity when used for 3D printing ink. Excess conductive filler will meet the requirement of conductivity but cause the material viscosity to be too high, resulting in the phenomenon of printing nozzle blockage. Inversely, the conductive ink can meet the rheological characteristics of printability but often fails to meet the required conductivity.

According to the material properties, the 3D printing used to manufacture flexible sensors mainly comprises direct ink writing (DIW), fused deposition modeling (FDM), digital light processing (DLP), stereo lithography (SLA), and material jet printing (MJP). Table 2 summarizes the printable materials, forming accuracy, and advantages and disadvantages of these five techniques.

**Figure 9:**
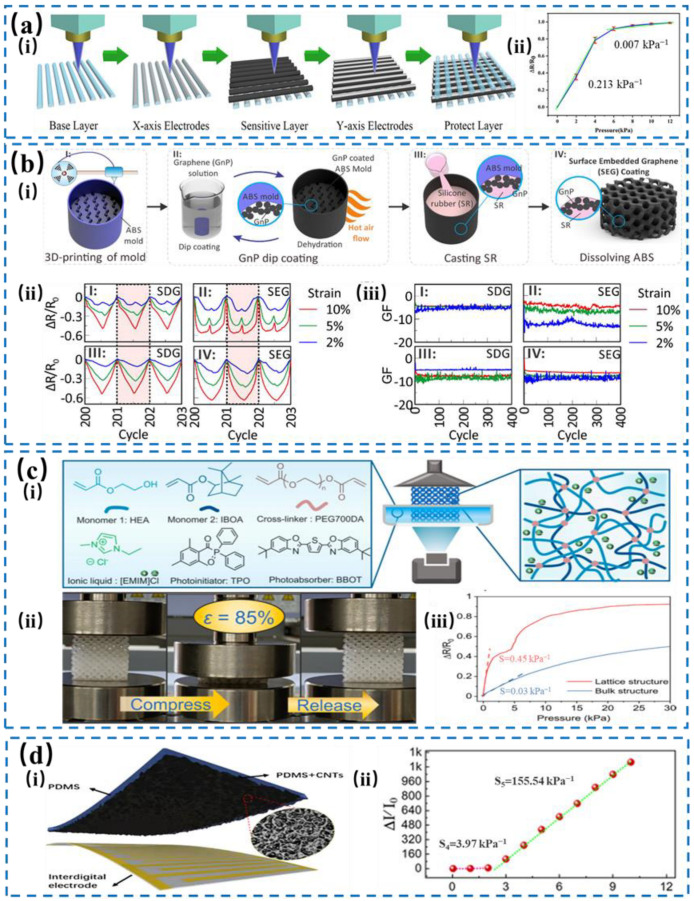
(**a**) Flow chart of the fabrication of e-skin 3D printing based on a porous ionic gel (i) with a high sensitivity of 0.213 kPa^−1^. (ii) Pressure vs resistance response of the e-skin pressure sensor. Reproduced with permission from Ref. [100]. Copyright 2022, American Chemistry Society. (**b**) Surface-embedded graphene sensors. (i) SEG sensors are fabricated in the following procedures: An ABS mold was made via 3D printing and then was dip coating in the graphene solution repeatedly. After dehydration by hot air flow, graphene nanoplatelets (GnP) was adhered to the surface of ABS mold uniformly. Subsequently, silicone rubber (SR) was cast, leading to GnP transfer to the SR surface after dissolving ABS mold with resistance changes (ii) and stable gauge factors (iii) when applying 200 cycles and 0~400 cycles under strain of 2%, 5%, 10%. Reproduced with permission from Ref. [101]. Copyright 2020, American Chemistry Society. (**c**) Schematic illustration of DLP 3D printing for wearable devices with different structures (i). (ii) The image of the compression/release state of the lattice-structure sensor under 85% strain. (iii) The pressure sensitivity when sensors are lattice-structured or bulk-structured. Reproduced with permission from Ref. [102]. Copyright 2022, Elsevier. (**d**) The structure design of the sandpaper morphology and PDMS/CNTs (SPC) sensor consisting of the SPC film and interdigitated electrode layers (i). (ii) The sensitivity of the 600-mesh-roughness SPC sensor within the range of 10 kPa. Reproduced with permission from Ref. [103]. Copyright 2022, American Chemistry Society.

DIW can extrude viscoelastic colloids and their composites through the print head and manufacture the required 3D structure in a layer-by-layer accumulation manner, such as planar capacitive pressure sensors and stretchable electrodes. Zhang et al. [100] used direct 3D printing to prepare a porous ionic gel flexible sensor containing ionic liquids (ILs) and acrylate monomers, which achieved a high sensitivity of ~0.213 kPa^−1^, as shown in Figure 9a. For better breathability, the spacing among fibers was 400 μm, which is 5 times less than that of hair follicles on the human arm, by controlling the printing fill rate. In comparison to DIW, FDM can produce composite multiple functional materials during the printing process to achieve multi-material structures via as-equipped screw-driven filament extrusion and direct melt particle extrusion. In Figure 9b, Toyserkani et al. [101] used 3D printing acrylonitrile butadiene styrene (ABS) material porous mold to realize the honeycomb-porous-structure pressure sensor with a graphene oxide coating, which presented an electrical resistance durability of 12 months and high tolerance for organic solvents. For properties, this pressure sensor retained favorable conductivity and strain recoverability under severe conditions with a gauge factor of 10 within the compressive strain range of 2~10%.

Both SLA and DLP are based on the polymerization of photosensitive polymer under ultraviolet, in which SLA uses a laser source to directly write on the surface of the polymer, while DLP employs digital light projection to solidify the pattern of each layer. Figure 9c illustrates a photo-curable resin containing ionic liquids and acrylate monomers [102]. Through DLP, customized porous ionic gel flexible sensors were prepared, which exhibit a high pressure sensitivity and lower hysteresis of 2.4% due to the complex porous structure composed of organic frameworks, hollow footballs, and various lattice structures, and can provide stable signals during long-term loading of >500 cycles. Material jetting, also known as “Multi-jets” technology, is one hybrid technique of inkjet printing and photo-polymerization. This method uses a multi-channel dispenser to distribute droplets of photo-polymerizable material according to the layer configuration defined by the CAD model and is solidified by a UV light source. As shown in Figure 9d, Yang et al. [103] prepared a film piezoresistive sensor based on the microstructure of sandpaper surface. Combining inkjet printing with the self-diffusion process of carbon nanotubes, the conductivity of traditional CNT-doped conductive ink was refined, which resulted in a sensitivity of 155.54 kPa^−1^, an ultrawide pressure sensing range from 0.1 kPa to 500 kPa, and an exceptional durability of over 12,000 pressure cycles at 300 kPa. Therefore, MJ technology can also be a potential method to develop multifunctional flexible wearable sensors. Table 2 outlines the various aspects on the types of 3D printing techniques with the corresponding materials, resolutions, advantages, and disadvantages.

**Table 2:** Lists of types of 3D printing with materials, resolutions, advantages, and disadvantages.

Types	Materials	Resolution	Advantages	Disadvantages	Ref.
DIW	Polymer, e.g., hydrogel Electronic materials, e.g., semiconductor and conductor	1~100 μm	High resolution Material versatility Customization and complexity	High equipment costs Low printing speed Material limitations Low durability and longevity	[104,105,106]
FDM	Thermoplastic materials, e.g., cellulose nanocrystals and polymer composites	50~400 μm	Material accessibility Ease of use Structural integrity Customization and flexibility Rapid prototyping	Limited resolution Material limitations Printed layer visibility Potential for warping and shrinkage	[107,108,109]
DLP	Photopolymer and its composites	25~100 μm	High resolution Smooth surface finish Rapid prototyping Material versatility Consistency	Limited build volume Material cost and availability Post-processing Sensitive to light and storage conditions	[110,111,112,113]
SLA	Photopolymer and its composites	25~100 μm	High resolution Smooth surface finish Material versatility Consistency High elaboration of detail	Limited build volume High cost for material, operation and maintenance Post-processing Limited durability	[114,115,116,117,118]
MJP	Photopolymer and thermoplastic materials	<300 μm	High resolution Multi-material printing Smooth surface finish High precision and consistency Efficient use of materials	High material cost Post-processing Limited durability Limited build volume	[119,120,121]

Three-dimensional (3D) printing technologies, like DIW (Direct Ink Writing), FDM (Fused Deposition Modeling), DLP (Digital Light Processing), SLA (Stereolithography), and MJP (MultiJet Printing), offer unique advantages and limitations. DIW excels in printing viscous materials with moderate resolution. FDM is widely used for its cost-effectiveness and material variety but lacks fine resolution. DLP and SLA provide high-resolution and smooth surface finishes, with SLA being particularly noted for its precision in intricate details, but both can be limited by material compatibility and post-processing requirements. MJP offers high resolution and the ability to print in multiple materials simultaneously, though it can be costly and involves extensive post-processing.

### 3.3. Four-Dimensional (4D) Printing

Four-dimensional (4D) printing, evolved from 3D printing, refers to one process of creating 3D objects that can transform over time in response to environmental stimuli, such as light, heat, and moisture. Four-dimensional printing is an important trend for tactile sensors, which can control the spatial position of different materials. Nowadays, the novel functions realized by 4D printing are mainly concentrated in the aspects of self-healing, integrated sensing and driving, and self-adaption. For example, Hao et al. [43] fabricated self-healing triboelectric nanogenerators or TENGs with an output power density of 56 mW/m^2^ based on shape memory polymer via 4D printing, which exhibits excellent self-recovery ability and robustness. However, the nature of shape memory polymers limits their adaptability to unknown environments when perceiving outside stimuli. Herein, Leng et al. [122] synthesized PLMC/CNT nanocomposite material with electric-induced deformation ability for 4D printing. As-printed deformable liquid sensors based on the composite printing ink could self-adapt to different liquid levels with high precision. This self-adaption could be used in aerospace, robotics, and other fields to tackle complex environmental adaptability. In addition, ink composed of conductive fillers with stimulus-responsive smart materials could realize the self-sense for motion, position, and posture, which is one of the hot topics relevant to 4D printing. Shi et al. [123] reported a 4D-printed high-performance integrated sensor-actuator with biomimetic microstructure, which showed a gauge factor of 48.46 and a high-temperature sensitivity of about 1.9~2.25 × 10^4^ ppm/°C. In terms of applications, the potential orientations of 4D-printed sensors are mainly to enhance the detection of various physiological signals of biological bodies, and to improve the performance, function, adaptability, and durability of sensors via fine design.

### 3.4. Transfer Printing

Transfer printing is an emerging technology that transfers nano/micro devices from donor substrates to recipient substrates, which is of great significance for the development of flexible electronics that require heterogeneous inorganic electronic materials and soft substrates [124,125,126]. The typical printing process includes pick-up and printing. In the pick-up step, the functional devices that are previously fabricated on the donor substrates, such as micro/nano films, ribbons, nanowires, nanotubes, etc., are picked up to the flexible film [127,128]. In the printing step, the inked stamp contacts the recipient substrate, and then the stamp is removed to release the devices onto the recipient substrate. The prerequisite for a successful pick-up step is that the adhesion strength at the stamp/device interface is greater than that at the device/donor interface, resulting in delamination at the device/donor interface and thereby transferring to the elastomer stamp [129,130,131]. For the printing step, the adhesion strength at the device/receiver interface is stronger than that at the stamp/device interface, thus enabling printing [132,133,134]. In order to realize a high-performance ultrathin tactile device, Niroui et al. [135] reported one transfer method to decouple the forces at the interface of van der Waals (vdW) layers and Si substrate via a hybrid high- and low-adhesion surface, which retained intrinsic physical properties of 2D materials. Although the transferring process of a single device has been realized, batch transfer still sets blocks for the large-scale production of 2D tactile devices. Di et al. [136] developed a graphene-assisted metal transfer printing process to overcome chemical disorder and Fermi-level pinning via conventional metal deposition, which enabled metal electrode arrays with weak (Cu, Ag, Au) and strong (Pt, Ti, Ni) adhesion strengths to be transferred from a four-inch graphene wafer onto recipient substrate due to weak vdW force and absence of dangling bonds. Kim et al. [137] designed a high-throughout layer transfer technique, which was used for production of multi-compound 2D membranes from a single wafer. A time efficiency that avoids sacrificial layer etching and wafer polishing, and atomic-precision exfoliation for membrane production was achieved. Transfer techniques open a novel path to reduce costs and realize batch production for heterogeneously integrated tactile devices in the future.

## 4. Representative Applications of Tactile Sensors

Tactile devices and systems have expansive application potential, especially in intelligent robotics, wearable devices, prosthetics, etc. In robotics, neuromorphic tactile sensors are used to help robots grasp and manipulate objects with more dexterity and precision, as well as to interact safely with humans and the environment. For wearable devices, neuromorphic tactile sensors can be integrated into skin-conformable devices that can monitor the physiological and environmental signals of the wearer, such as pressure, temperature, humidity, and motion. In prosthetics, neuromorphic tactile sensors can provide sensory feedback to amputees, which can enhance their sense of embodiment and improve their motor control and functionality.

### 4.1. Intelligent Robotics

**Figure 10:**
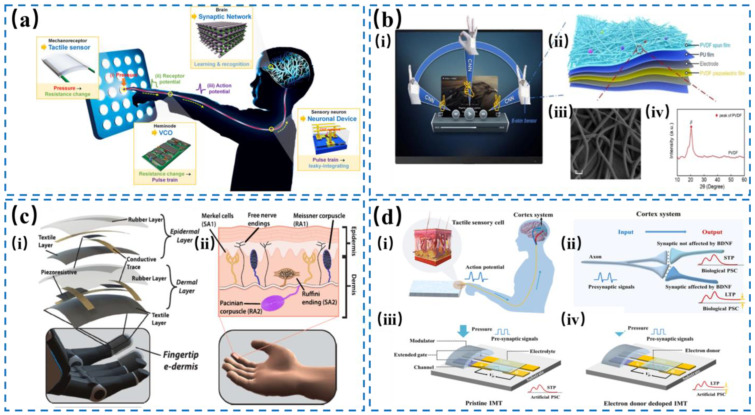
(**a**) Schematic illustration of a robotic tactile system, in which pressure stimuli applied to mechanoreceptors induce changes in the receptor potential and then initiate action potentials. Action potentials activated by multiple nerve fibers are superposed through neurons and used for information processing, resulting in the synaptic network of the brain recognizing the input pressure pattern. Reproduced with permission from Ref. [1]. Copyright 2020, Springer Nature. (**b**) Schematic illustration of gesture recognition and control of the tactile sensor system (i). (ii) The structural design of the tactile sensor. (iii) SEM image of PVDF nanowires prepared via electrospinning with a scale bar of 5 μm. (iv) XRD pattern of PVDF film. Reproduced with permission from Ref. [143]. Copyright 2022, American Chemistry Society. (**c**) The structural diagram of the multilayered e-dermis over the fingertips of a prosthetic hand (i), including conductive and piezoresistive textiles encased in rubber, in which a dermal layer of two piezoresistive sensing elements is separated from the epidermal layer with a silicone rubber layer of 1 mm. (ii) The counterparts of humans’ biological mechanoreceptors in healthy glabrous skin. Reproduced with permission from Ref. [144]. Copyright 2018, Science. (**d**) The procedures that a biological system uses to perceive (i) and process (ii) external pressure. Schematic diagram of the volatile pristine in-memory tactile sensor (iii) and the nonvolatile PEI de-doped in-memory tactile sensor (iv). Reproduced with permission from Ref. [145]. Copyright 2023, American Chemistry Society.

Intelligent robotics have been extensively explored for their ability to solve complex problems relevant to humans, such as interacting with human beings in a friendly manner, identifying unknown objects delicately [51,61], and tackling tasks in unstructured environments with dexterity and adaptability. Over the past years, many researchers have devoted themselves to the continuous advancement of more intelligent robotics. Tactile perception may be one of the grand challenges that remains to be addressed for intelligent robotics, and neuromorphic devices could be one possible solution for this. As shown in Figure 10a, Choi et al. [1] developed an artificial tactile sensor system that could emulate the human sensory system’s ability to detect and learn from patterns of stimuli. The system consisted of a semi-volatile CNT transistor device that acted as a sensory neuron and a synaptic network, and it could improve its recognition accuracy of tactile patterns through an iterative learning process. In order to perceive multiple things using a tactile sensor, Xu et al. [138] created a device called a nanowire-channel intrinsically stretchable neuromorphic transistor (NISNT) that could sense touch and vision and mimic brain-like functions. It could stretch up to 1000 times without losing its electrical properties and be used as a nerve that processes different types of information simultaneously. This device can be attached to fingers conformally to measure the strain and movement of the fingers, and then analyze the signals and recognize different gestures via a neural network. In addition, Sun et al. [139] proposed a multifunctional e-skin that mimicked the chameleon’s ability to change color according to the pressure it feels, based on two types of self-powered devices: triboelectric sensor (TES) arrays and electrochromic device (ECD) arrays. The TES arrays measured the pressure at different parts of the robot, and the ECD arrays showed the pressure by changing the color of the e-skin. The e-skin could provide real-time tactile and visual feedback for the robot and the object it touched. It could also fit large and curved robot surfaces because of its flexibility and scalability. Neuromorphic tactile sensors are a novel and promising way to improve human–robot interactions with dynamic tactile feedback systems. To reproduce the tangential “sliding” perception of human skin, Shao et al. [140] introduced a bioinspired robot skin capable of ultrasensitive and fast-response sliding tactile perception. This innovative skin, featuring mechanically gated electron channels within a monolithic structure of CNT networks and PDMS, mimicked the lateral gating sensing mechanism of human tactile sensory cells. The R-skin could detect ultrafine surface textures with a high accuracy of 5 μm and a speed of 485 Hz, outperforming human tactile capabilities. This development opens new avenues for robotic tactile sensing with potential applications in intelligent robotics and human–machine interfaces by providing detailed tactile feedback and enhancing interaction with the environment. Zhu et al. [141] introduced a skin-inspired quadruple tactile sensor integrated onto a robotic hand, enhancing object recognition through grasping. This sensor combined pressure, material thermal conductivity, and temperature sensing to achieve precise identification of object shapes, sizes, and materials, demonstrating a 94% accuracy in garbage sorting applications. Kim et al. [142] reported a bioinspired robot skin (R-skin) capable of ultrasensitive and fast-response sliding tactile perception, mimicking human tactile sensory cells, and featuring a novel design using carbon nanotube networks and polydimethylsiloxane. The R-skin enabled ultrafine texture detection with a detection limit of 0.7 N, which surpassed human tactile capabilities, promising advancements in robotic tactile sensing and interaction.

### 4.2. Wearable Devices

Wearable tactile devices can provide a more natural and realistic touch sensation for the wearer and are used to improve immersion in virtual reality/augmented reality (VR/AR) systems [146,147,148,149,150]. The glove is the bridge connecting virtuality and reality, and is used to send real-time tactile-feedback information. However, some drawbacks still block timely feedback, such as simple vibration, narrow bandwidth, and single tactile perceptions. In order to address these problems, Lee et al. [151] developed a smart low-cost glove that could sense and stimulate touch for virtual interaction, as shown in Figure 10b, employing triboelectric sensors to measure finger bending and palm sliding and piezoelectric stimulators to provide tactile feedback. It could detect different hand movements and gestures in virtual space and could also recognize objects using machine learning with a high accuracy of 96%. Moreover, Li et al. [143] developed a wearable sensor that could sense and control gestures using deep learning, made of two types of self-powered devices that generate electricity from touch. It had high power density with 0.35 mW cm^–2^ and sensitivity with fast response of 5 ms, low noise of ~22.5 dB, and fine pressure resolution of 1% (1~10 kPa). Although researchers have achieved giant advances in wearable tactile systems, they still face challenges in creating realistic and immersive VR/AR experiences. In order to enhance the interconnection between augmented and virtual reality, Matusik et al. [152] presented a novel approach to enhance tactile interaction transfer through digitally embroidered smart gloves, which were integrated with tactile sensors and vibrotactile tactile actuators, allowing for the capture, reproduction, and transfer of tactile interactions. Furthermore, a machine-learning pipeline was developed to adaptively model individual users’ responses to tactile feedback and optimize the feedback parameters. The research showcases applications in alleviating tactile occlusion, guiding physical skill performance, and enhancing robot teleoperation. Lee et al. [153] introduced augmented tactile-perception and tactile-feedback rings (ATH-Rings) designed to enhance VR experiences, featuring triboelectric and pyroelectric sensors for tactile and temperature sensing, along with vibrators and nichrome heaters for vibro- and thermo-tactile feedback. Integrated into a compact, wireless platform, the rings aimed to provide immersive interactions in VR applications by enabling real-time, continuous finger motion tracking and gesture and object recognition through artificial intelligence analysis and interactive metaverse platforms. This innovative approach offers a new dimension to human–machine interfaces, promoting immersive virtual social experiences. In addition to smart gloves, e-skin is also a ground-breaking strategy for sensing imperceptible outer changes. Song et al. [154] discussed a multifunctional electronic skin based on patterned metal films designed for tactile sensing, capable of detecting pressure and temperature simultaneously with a broad linear response range of 80 kPa. This e-skin utilized a single sensing mechanism of piezoresistance, simplifying signal processing and device configuration. Through experimental and numerical studies, the e-skin’s design and operation principle were explored, demonstrating its flexibility and wearability. More importantly, the scalable manufacturing process aligns with existing microfabrication techniques, promising for various practical applications.

### 4.3. Prosthetics

Amputees can receive stimuli from pressure and vibration signals via tactile sensors. However, amputees still need to endure a signal delay in view of the timeliness and accuracy of microprocessors in prosthetics [1,147]. So far, researchers have done tremendous work to increase the levels of sophistication of prosthetics. For example, Wang et al. [155] showed a new type of piezoresistive memristor for prosthetics that could switch between digital and analog modes through sensing mechanical stimuli and act as an artificial synapse for neuromorphic computing, which was based on CsPbBr_3_ material with an on/off ratio of 10^3^, a retention time of 10^4^ s, an endurance of ~100 cycles, and multilevel resistance. Furthermore, as shown in Figure 10c, Thakor et al. [144] reported a layered e-skin for a prosthetic hand and its user to sense touch and pain, depending on how our body’s receptors and nerves work and how amputees perceive pain or no pain in their missing hand via brain activity. The user could tell apart painful or nonpainful touches and judge the shape and sharpness of objects through a series of touch and pain signals from e-skin. These works showcased the potential to generate a more lifelike experience by encompassing various tactile stimuli in the field of prosthetics. To endow manipulators or prosthetics with artificial tactile perception, fingers are the key components of prosthetic limbs to realize the identification for materials and surfaces of things. Wang et al. [156] introduced a smart finger that surpassed human tactile perception, capable of accurately identifying material types and their roughness through a combination of triboelectric sensing and machine learning. The device picked out different materials via the unique triboelectric fingerprints upon contact, achieved by an array of triboelectric sensors to minimize environmental interference. With a high accuracy rate of 96.8% in material identification, this smart finger offers potential enhancements for manipulators or prosthetics, providing them with artificial tactile perception capabilities. To enhance sensory feedback and motor control for amputees, Micera et al. [157] reported the development and clinical implementation of a transradial neuromusculoskeletal prosthesis that integrated directly with the user’s nervous and skeletal systems. This bionic hand, attached via titanium implants and equipped with electronic muscle, offered meliorative prosthetic functionality with a high accuracy of 75.5% and reduced post-amputation pain through reliable neural control and feedback. Frequent use in daily activities demonstrates its effectiveness and potential to overcome limitations of conventional prosthetics, highlighting significant advances in prosthetic technology and human–machine interfaces. Guglielmelli et al. [158] described a closed-loop hand prosthesis providing simultaneous intraneural tactile and position feedback to transradial amputees. By employing implanted electrodes and sensory substitution, the prosthesis enabled amputees to regain high levels of proprioceptive acuity and tactile feedback, allowing for improved object discrimination. The integration of this feedback significantly enhanced the functionality and embodiment of the prosthesis, showcasing a promising advance towards more sophisticated bionic limbs that convey richer multimodal sensations and improve the quality of life for amputees. Branemark et al. [159] showcased a study on restoring tactile sensations and enabling real-time force-and-slippage control in bionic hands through neural interfaces, thus detailing the development of a system that provided amputees with close-to-natural sensations of force and slippage, significantly improving manipulative skills with the prosthesis. This approach used a combination of cuff and intraneural electrodes, demonstrating substantial enhancements in grasp and manipulation tasks, supported by quantifiable improvements in motor performance and sensory feedback, which underlined the potential of neural interfaces in prosthetic control and sensory restoration.

### 4.4. Health Care

Biomechanical information, such as pulse, blood pressure, intraocular pressure, and articular movement, is pivotal to monitor and measure patients with chronic and acute diseases and assess their health status after reconstructive surgeries [160,161,162,163,164]. To address the coupling relationship between adaptive receptors and artificial neurons, Hao et al. [165], by integrating a polypyrrole-based pressure sensor with a volatile NbOx memristor, designed an artificial mechanoreceptor that could mimic skin-like tactile sensation and perception. This mechanoreceptor was capable of encoding external mechanical stimulus into electrical spikes and enhancing tactile sensation through processing spike frequency signals using a pulse-coupled neural network, and could promote the development of future bio-inspired electronic systems. In addition, to fulfill the requirements of sensing complex information exactly, Chen et al. [166] and Zhang et al. [167] developed a chemically mediated artificial neuron based on a carbon-based electrochemical sensor to detect dopamine and a synaptic memristor to process the sensory signals, and realized a stretchable temperature-responsive multimodal neuromorphic e-skin by integrating stretchable neuromorphic transistors with mechano- and thermos-receptors to conduct sensory and memory functions, respectively. However, conventional methods turn to integrating multi-sensory modalities to guarantee accuracy, sensitivity, and effectiveness, which will increase the complexity of circuits, energy consumption, and crosstalk among signals. As shown in Figure 10d, Hu et al. [145] proposed an in-memory tactile sensor (IMT) with a programmable steep-slope region and retention time of >1000 s based on a mechano-gated transistor. In view of programmability and nonvolatility, the IMT could realize sensing signals on demand, mechanical cue mapping with high spatiotemporal resolution, and associative learning between two physical inputs, leading to precise assessment for patients’ health status with an ultralow power dissipation of ~25.1 μW. In addition, minimally invasive surgery (MIS) and robotic minimally invasive surgery (R-MIS) face challenges in tactile feedback due to reduced physical sensation from surgery. Innovations in tactile technology, such as gloves equipped with soft robotic sleeves and actuators, offer solutions by transmitting tactile feedback from the interaction between surgical instruments and tissues directly to the surgeon’s hands. Russo et al. [168] presented a proof-of-concept soft robotic glove that provided tactile feedback to the surgeon’s hand during interventional endoscopy procedures, specifically colonoscopy. The glove with pneumatic actuators was connected to a force sensing soft robotic sleeve that was mounted onto a colonoscope. When sensing the incident forces, the glove is capable of alerting the surgeon of potentially dangerous forces exerted on the colon wall by the colonoscope during the navigation. The glove was calibrated to respond to incident forces on the soft robotic sleeve ranging from 0 to 3 N with an internal pressure of 53 kPa and exerted forces up to 20 N, thereby relaying and amplifying the force exerted by the colonoscope on the colon to the surgeon’s hand.

## 5. Challenges and Perspectives

Recent years have witnessed remarkable progress in the development of tactile sensors based on distinctive structures, mechanisms, and fabrication techniques, and fruitful results have been achieved. Moreover, tactile sensory systems based on flexible synaptic devices have been constructed to mimic certain bio-inspired functions. With the ongoing advancement of neuromorphic devices, artificial tactile systems will potentially contribute to long-term developments in human–machine interaction. Neuromorphic tactile devices can be used in intelligent robotics to enhance perceptive capability in the future. For example, neuromorphic tactile hardware can likely help robotics adapt to less-constrained natural environments [169], and process external information more efficiently, and transmit it to the brain more rapidly than conventional sensors. Although neuromorphic tactile sensors have been involved in many aspects of HMI, such as robotics [170,171,172,173], wearable devices [40,174], prosthetics [175,176], and healthcare [166,167], the as-mentioned technologies are still in the initial stage of development and far from practical applications. Several key challenges relevant to neuromorphic tactile systems remain to be addressed and are discussed below.

### 5.1. Power Consumption of Tactile Sensors

Power consumption is a core indicator to evaluate the quality of artificial tactile systems based on synaptic sensors. Generally, the human neural network consists of about 10^12^ neurons and 10^15^ synapses, and each synapse just costs 1~10 fJ [177]. By contrast, power consumption per artificial synapse is larger than that of the biological counterpart. In order to reach this target, many researchers are devoted to reducing the power consumption of neuromorphic tactile sensors from the aspect of materials and structures. Currently, 2D materials such as graphene [178,179,180,181,182], transition-metal dichalcogenides [183,184,185,186,187,188], and black phosphorus [189,190,191,192], are emerging as platforms to be constructed into tactile and synaptic sensors with low power consumption, low working voltage, and high flexibility. In addition, special structures can help improve sensing performance. For example, Xing et al. [193] proposed one octopus-inspired tactile sensor with a sensitivity of 0.636 kPa^−1^, response time of ~40 ms, and durability of >6000 cycles, validating it as a reliable platform for us in intelligent robotics and e-skins. Current flexible tactile sensors and bionic perception systems based on them still exhibit significant gaps in power efficiency when compared to their biological equivalents, highlighting the urgent need for continued research and development aimed at minimizing power consumption in both the devices and the overall systems. For example, the adoption of innovative, low-power, two-dimensional materials in material selection could play a crucial role. Similarly, the exploration of three-dimensional fabrication techniques for flexible tactile sensors could pave the way for more efficient designs. Additionally, the development of self-powered tactile sensing systems presents a promising approach to reducing power consumption. Specifically, systems utilizing piezoelectric or triboelectric principles can generate electrical energy from mechanical motions, such as pressure or friction, eliminating the need for external power sources. Piezoelectric tactile sensors convert mechanical stress into electrical signals, offering a direct method for energy harvesting and sensing, which can significantly enhance power efficiency. On the other hand, triboelectric sensors harness the electrical charge generated from contact and separation processes between two different materials, providing a unique, low-power solution for tactile sensing. Incorporating these self-powered technologies into tactile sensing systems could drastically decrease their energy requirements, making them more comparable to the efficiency found in natural biological systems.

### 5.2. Stability and Endurance of Tactile Sensors

Long-term stability is crucial for the consistency of neuromorphic tactile sensors, which determines their sensitivity and accuracy over time, and under various environmental conditions, such as temperature and humidity. More and more researchers are focusing on increasing the stability and endurance of neuromorphic tactile sensors through selecting rational materials, delicate structures, and reliable fabrication processes. For example, Chien et al. [194] observed a steady and reliable sensing capability under the aid of a porous dielectric framework and conducting nanowires over 1000 pressure load/unload cycles with a response time of 100 ms and recovery time of 100 ms. As another example, Kim et al. [195] introduced the hierarchical sea-urchin TiO_2_ particle-in-micropore structure into a dielectric layer via a mixed solvent phase separation method, which showed a pressure sensitivity of 10.5 kPa^−1^ at 100 Pa and response/relaxation times both of 5.6 ms after 10,000~12,000 compression/bending cycles. Currently, the majority of research efforts in the field are focused on proof-of-concept studies, with limited efforts aimed at improving the stability and endurance of devices and systems. Yet, enhancing both stability and durability is essential for their practical application in the future. One effective strategy to boost device stability involves the use of encapsulation materials that protect against environmental factors. Moreover, adjusting the chemical and mechanical properties of materials to create ones that inherently possesses long-term stability is a promising avenue. Specifically, incorporating microporous materials such as Titanium Dioxide (TiO_2_) could offer significant improvements. TiO_2_ is known for its robust chemical stability, photocatalytic properties, and biocompatibility, making it an excellent candidate for enhancing the durability of tactile sensors. By leveraging such materials, alongside innovations in material science, future research undertakings can lead to tactile sensors with superior long-term stability and endurance. These advancements are particularly relevant for applications in robotics and prosthetics, where reliability and longevity are paramount, allowing for more effective and dependable use.

### 5.3. Feedback Time of Tactile Sensors

The speed at which these sensors process and respond to tactile information is vital, especially in applications requiring real-time interaction, like robotics or prosthetics. Generally, the feedback time of a human varies from a few milliseconds to several milliseconds. Minimizing the feedback time without sacrificing accuracy is a significant challenge. Researchers are focusing on utilizing innovative approaches and technologies to enhance the responsiveness and efficiency of tactile sensors. Inspired by human somatosensory feedback pathways, Zhang et al. [196] constructed an In_2_O_3_-based artificial somatosensory system with spatiotemporal information processing ability via integrating tactile sensors and synaptic transistors, showing a feedback time of 50~200 ms. Currently, tactile sensing systems are still grappling with critical challenges in feedback timing, such as latency in signal processing and delayed responses to stimuli, hindering their effectiveness in real-time applications. To mitigate these issues, a multifaceted approach is recommended. This includes optimizing the sensor’s architecture to facilitate faster signal transmission, refining computational algorithms for more rapid data processing, and employing advanced materials that respond more swiftly to stimuli. Specifically, materials like Indium Oxide (In_2_O_3_), known for its high charge carrier mobility and saturation velocity, can significantly enhance sensor reactivity, offering a substantial leap in performance. Additionally, improving the synergy between the hardware and software of the sensor systems can greatly reduce feedback times. Such advancements will render tactile sensors far more suitable for applications that demand immediate responses, such as robotic control and interactive technologies, by ensuring quicker and more reliable sensory feedback.

## Figures and Tables

**Table 1:** Comparison of resistive-, capacitive-, piezoelectric-, and triboelectric-typed tactile sensors.

Sensor Types	Sensor Features	Sensitivity	Sensing Range	Applications	Ref.
Resistive tactile sensors	Multi-walled carbon nanotubes	~385 kPa^−1^	>1400 kPa	smart robotics	[54]
Resistive tactile sensors	CNT/Au electrode with a pyramid-structure resistive channel	/	1~80 kPa	neurorobotics and neuro-prosthetics	[56,85]
Resistive tactile sensors	Al_2_O_3_/MoS_2_/Al_2_O_3_ sandwich structure	0.011 kPa^−1^	1~120 kPa	wearable electronics, e-skin, bio-robotics	[86]
Capacitive tactile sensors	AgNF-AgNW hybrid electrode	1.78 × 10^−3^ kPa^−1^	<350 kPa	mobile smart devices	[35]
Capacitive tactile sensors	Graphene/CNT/Silicone rubber composite	0.63 kPa^−1^	0.1~0.26 MPa	robotic prostheses	[44]
Capacitive tactile sensors	Au/ polystyrene (PS)/Au layer with micropatterned PDMS	0.815 kPa^−1^	0~50 N	electronic skins, wearable robotics, and biomedical devices	[87]
Capacitive tactile sensors	PDMS/microconformal graphene (MGr) structure with PET substrate	3.19 kPa^−1^	0~4 kPa	wearable health-monitoring devices, robot tactile systems and human-machine interface systems	[59]
Piezoelectric tactile sensors	Polyacrylonitrile/barium titanate (PAN-C/BTO) nanofiber film	1.44 V·N^−1^	0.15~25 N	human-computer interactive and smart wearable sensing systems	[88]
Piezoelectric tactile sensors	PVDF nanofibers based on polyurethane (PU) film and PDMS plate	7.1 mV·kPa^−1^	<10 kPa	electronic skin, robotics, and interface of artificial intelligence	[89]
Piezoelectric tactile sensors	PVDF/BaTiO_3_ nanocomposites	18 V·N^−1^	1~20 g	human–machine interfaces	[90]
Triboelectric tactile sensors	Ecoflex electrification layer with polyvinyl alcohol/polyethyleneimine (PVA/PEI) electrode layer	0.063 V·kPa^−1^	5~50 kPa	self-powered touch screens, human–machine interfaces	[91]
Triboelectric tactile sensors	Micro-pyramid-patterned double-network ionic Organohydrogels	45.97 mV·kPa^−1^	0.02~4 kPa	wearable devices and robotics	[92]
Triboelectric tactile sensors	Carbonyl Iron powder (CIP)/NdFeB/PDMS Magnetic Composite and CNT/PDMS Mixture	0.314 kPa^−1^	>1000 kPa	healthcare monitoring	[37]

## Data Availability

Data are available upon request to the corresponding authors.
